# Tool-tissue forces in surgery: A systematic review

**DOI:** 10.1016/j.amsu.2021.102268

**Published:** 2021-03-31

**Authors:** Aida Kafai Golahmadi, Danyal Z. Khan, George P. Mylonas, Hani J. Marcus

**Affiliations:** a Imperial College London School of Medicine, London, United Kingdom; b HARMS Laboratory, The Hamlyn Centre, Department of Surgery & Cancer, Imperial College London, London, United Kingdom; c National Hospital for Neurology and Neurosurgery, London, United Kingdom; d Wellcome/EPSRC Centre for Interventional and Surgical Sciences, University College London, London, United Kingdom

**Keywords:** Tool-tissue interaction, Operative forces, Surgical forces, Force feedback, Force sensors, Haptic feedback, Smart instruments, Robotics, Robotic surgery

## Abstract

**Background:**

Excessive tool-tissue interaction forces often result in tissue damage and intraoperative complications, while insufficient forces prevent the completion of the task. This review sought to explore the tool-tissue interaction forces exerted by instruments during surgery across different specialities, tissues, manoeuvres and experience levels.

**Materials & methods:**

A PRISMA-guided systematic review was carried out using Embase, Medline and Web of Science databases.

**Results:**

Of 462 articles screened, 45 studies discussing surgical tool-tissue forces were included. The studies were categorized into 9 different specialities with the mean of average forces lowest for ophthalmology (0.04N) and highest for orthopaedic surgery (210N). Nervous tissue required the least amount of force to manipulate (mean of average: 0.4N), whilst connective tissue (including bone) required the most (mean of average: 45.8). For manoeuvres, drilling recorded the highest forces (mean of average: 14N), whilst sharp dissection recorded the lowest (mean of average: 0.03N). When comparing differences in the mean of average forces between groups, novices exerted 22.7% more force than experts, and presence of a feedback mechanism (e.g. audio) reduced exerted forces by 47.9%.

**Conclusions:**

The measurement of tool-tissue forces is a novel but rapidly expanding field. The range of forces applied varies according to surgical speciality, tissue, manoeuvre, operator experience and feedback provided. Knowledge of the safe range of surgical forces will improve surgical safety whilst maintaining effectiveness. Measuring forces during surgery may provide an objective metric for training and assessment. Development of smart instruments, robotics and integrated feedback systems will facilitate this.

## Highlights

• This review explores tool-tissue forces during surgery, a new and expanding field.
• Forces were lowest in ophthalmology (0.04N) and highest in orthopaedics (210N).
• Forces were lowest during sharp dissection (0.03N) and highest when drilling (14N).
• Being an expert (vs. novice) and having feedback mechanisms (e.g. haptic) reduced exerted forces.
• Development of force metrics will facilitate training, assessment & novel technology.

## 1 Introduction

The field of surgery is defined by the application of physical force in order to manipulate or incise tissue for the treatment of medical disorders. Surgical forces must be applied judiciously, in a precise and controlled manner, in order to carry out procedures efficiently without causing unnecessary tissue injury [[1], [2], [3]]. The discrimination between appropriate and excessive force is a skill acquired over years of rigorous training, with junior surgeons tending to apply more force than more senior colleagues [4,5]. Indeed, up to half of the technical errors made by surgical trainees are related to the use of excessive force [6]. The advent of working hours restrictions and minimally invasive surgical approaches (often more technically demanding with steeper learning curves) may add to the challenge for surgical trainees [7,8].

The measurement of forces applied during surgery at a tool-tissue level is an important step in developing solutions to this problem and improving the safety of surgery. Force measurement can provide a quantitative metric of surgical skills, potentially useful for surgical training and assessment [4,9,10]. Measurement also allows the definition and characterization of a safe range of forces for specific manoeuvre [1,4]. These data can be used to generate surgical simulations for training [11] and develop devices (e.g. robotic platforms and smart instruments) with force feedback mechanisms [[4], [12], [13], [14], [27]].

Thus, we sought to understand the forces applied at the tool-tissue level for a range of surgical specialities, tissues, and manoeuvres. We also assessed the impact of operator experience and force feedback, through a systematic review of the literature.

## 2 Methods

A systematic review of the literature was performed in accordance with PRISMA (Preferred Reporting Items for Systematic Reviews and Meta-Analyses) and AMSTAR (Assessing the methodological quality of systematic reviews) guidelines.

### 2.1 Search strategy and screening

Three databases were searched (EMBASE, MEDLINE and Web of Science) and using targeted search terms (Supplementary Table 1) as per the registered study protocol (PROSPERO ID: CRD42020170917). Articles (1950–2020) were searched on 12/05/2020 and duplicates were removed using Endnote ×9. Inclusion criteria were: 1) studies reporting any open or laparoscopic surgical procedure performed on humans, cadavers, animals and validated models; 2) studies that described tool-tissue interaction forces measured directly. The inclusion of a simulation/model was based on whether it had proven face validity, content validity and construct validity (in the index study or in previous and cited work). Exclusion criteria were: reviews, editorials, non-English papers, full-text unavailability, unvalidated simulations and studies where no specific force values were recorded. Independent abstract screening was performed in duplicate by two authors (DZK, AKG). Review of full-text articles ensued according to the inclusion/exclusion criteria. Any discrepancies in selection were settled out by discussion and mutual agreement.

### 2.2 Data extraction and analysis

Data extracted from each eligible study included study demographics (continent, journal type), model characteristics (human/animal/synthetic, tissue type), procedure characteristics (surgical speciality, surgical task, instrument used), operator features (number, level of expertise) and force-related information (force measurement techniques, force levels, force-related complications). Adult surgical specialities (cardiothoracic surgery, otolaryngology, general surgery, plastic surgery, trauma and orthopaedic surgery, urology, neurosurgery, maxillofacial surgery) were defined as per Royal College of Surgeon England recognition [15,16] with obstetrics and gynaecology added as an additional speciality. Tissues were classified according to the four primary tissue types (epithelial, connective, nervous, muscle) where available. Manoeuvres were categorized into: retraction without grasping (manoeuvring tissue while not within the jaws of an instrument), retraction with grasping (manoeuvring tissue within the jaws of an instrument), blunt dissection (separating tissue planes using the blunt end of an instrument), blunt penetration (advancement of a blunt object through tissue), sharp penetration (advancement of a sharp-tipped object through tissue), cutting (incising or dissecting tissue using sharp scissors or blade), coagulation (cauterising a vessel), clamping (occluding the lumen of tubular tissue through the application of external closing force), suction (clearing the surgical field using suction) and drilling (use of a rotary cutting tool to cut or shave solid tissues) [1].

In terms of data extraction, if study-level averages for average (mean/median) or max forces were reported, these were extracted and pooled. If experiment-level raw data was available, these were summarised via mean calculation and added to the overall pooled analysis. Summary statistics (mean of average, mean of maximum) with accompanying narrative synthesis were generated for measured forces per specialities, tissues and manoeuvres. For the impact of experience and feedback forces pooled means (mean of average) were presented relatively, as a percentage of the reference pool mean – e.g. pooled mean forces applied after feedback as a percentage of pooled mean forces without feedback. Risk of bias assessment was not undertaken owing to the experimental nature of the included papers.

## 3 Results

### 3.1 General characteristics

**Fig. 1:**
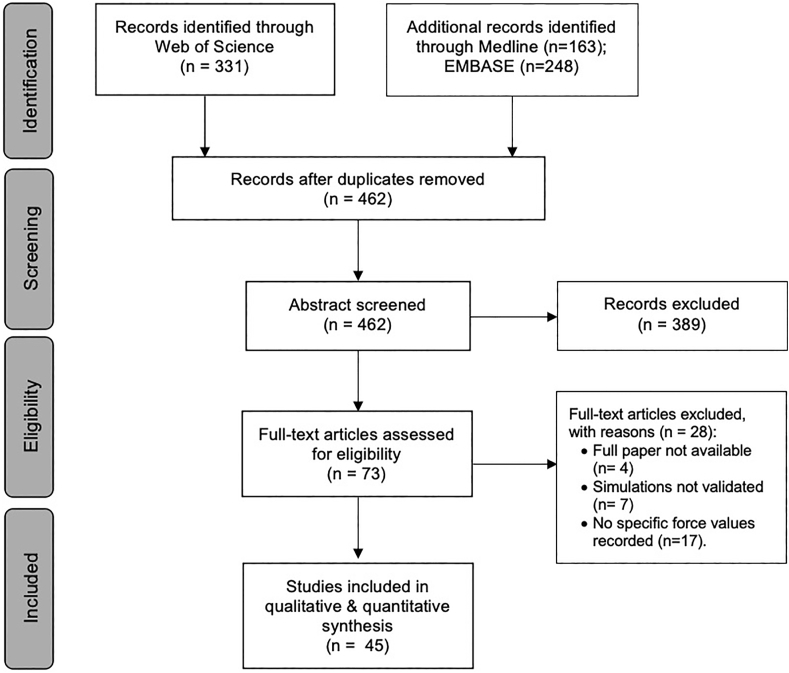
PRISMA flow chart of paper identification, screening and eventual inclusion.

Screening of 462 articles resulting in the full-text appraisal of 73 studies selected for full-text review. Ultimately, 45 studies met the inclusion criteria and progressed to narrative and quantitative synthesis (Fig. 1). In regard to study demographics, all studies were primarily conducted at North American (n = 19) [2,4,14,[17], [18], [19], [20], [21], [22], [23], [24], [25], [26], [27], [28], [29], [30], [31], [32], [33]] or European (n = 26) [1,3,5,[34], [35], [36], [37], [38], [39], [40], [41], [42], [43], [44], [45], [46], [47], [48], [49], [50], [51], [52], [53], [54], [55]] institutions (Supplementary Information 2). Articles were published in medical (n = 26) [[2], [3], [4],^14^,[21], [22], [23],^25^,^27^,^29^,^35^,^36^,^43^,[46], [47], [48], [49],^51^,^53^] or engineering (n = 19) [1,5,[17], [18], [19], [20],^24^,^26^,^28^,[30], [31], [32], [33], [34],[37], [38], [39], [40], [41], [42],^44^,^45^,^50^,^52^,^54^,^55^] journals between 1999 and 2020 (Supplementary Information 3). Evidently, this is a new and expanding field, with more articles published in the last 5 years (n = 24) [[2], [3], [4],^14^,^17^,^19^,^22^,^23^,^25^,^26^,^28^,[32], [33], [34], [35],^39^,^41^,[45], [46], [47],^50^,[53], [54], [55]] than the previous 25 years combined (n = 21) [1,5,18,20,21,24,27,[29], [30], [31],[36], [37], [38],^40^,[42], [43], [44],^48^,^49^,^51^,^52^] (Supplementary Information 3).

In terms of surgical specialty (Supplementary Information 2, Table 1), the highest proportion of studies (in-vivo and ex-vivo models) were in general surgery (n = 14, 31.1%) [3,5,20,26,27,35,38,[41], [42], [43],^49^,^51^,^54^] with the majority of these involving laparoscopic procedures (n = 11) [3,5,26,34,38,[41], [42], [43],^49^,^54^,^55^]; followed by neurosurgery (n = 13, 28.89%) [1,2,4,14,22,23,25,28,33,37,44,52], ophthalmology (n = 5, 11.11%) [[17], [18], [19],^21^,^32^], cardiothoracic (n = 3, 6.67%) [26,30,53], vascular surgery (n = 3, 6.67%) [29,40,50], obstetrics & gynaecology (n = 2, 4.4%) [39,45], orthopaedics (n = 2, 4.4%) [47,48], otorhinolaryngology (n = 2, 4.4%) [36,46] and urology (n = 1, 2.2%) [24]. Ten studies (22.2%) involved robotic surgery platforms [1,14,[17], [18], [19],^22^,^26^,^29^,^48^,^55^].

**Table 1:** Summary forces averages across speciality, tissue type surgical tasks.

Category	Mean of average (N)	Mean of maximum (N)	Number of studies
**Specialties**			
Ophthalmology	0.04	0.05	5
Vascular	0.07	0.65	3
Neurosurgery	0.68	1.48	13
Cardiothoracic	1.47	1.76	3
General surgery	4.67	11.4	14
Otorhinolaryngology	8.49	15.6	2
Obstetrics & gynaecology	8.69	10.1	2
Urology	9.79	15.6	1
Orthopaedics	210	1708	2
**Tissues**			
Nervous	0.4	1.7	17
Epithelial	3.8	9.7	18
Muscle	4.1	6.7	3
Connective	45.8	347.9	10
**Tasks**			
Sharp dissection	0.03	NA	1
Clamping	0.05	0.10	2
Suction	0.13	0.21	1
Coagulation	0.29	0.90	4
Retraction without grasping	0.95	2.51	3
Blunt penetration	1.21	3.58	4
Blunt dissection	2.08	3.69	7
Cutting	2.28	4.48	5
Sharp penetration	2.34	4.67	11
Retraction with grasping	3.65	10.25	13
Drilling	14.09	25.99	2

### 3.2 Summary forces for speciality

**Fig. 2:**
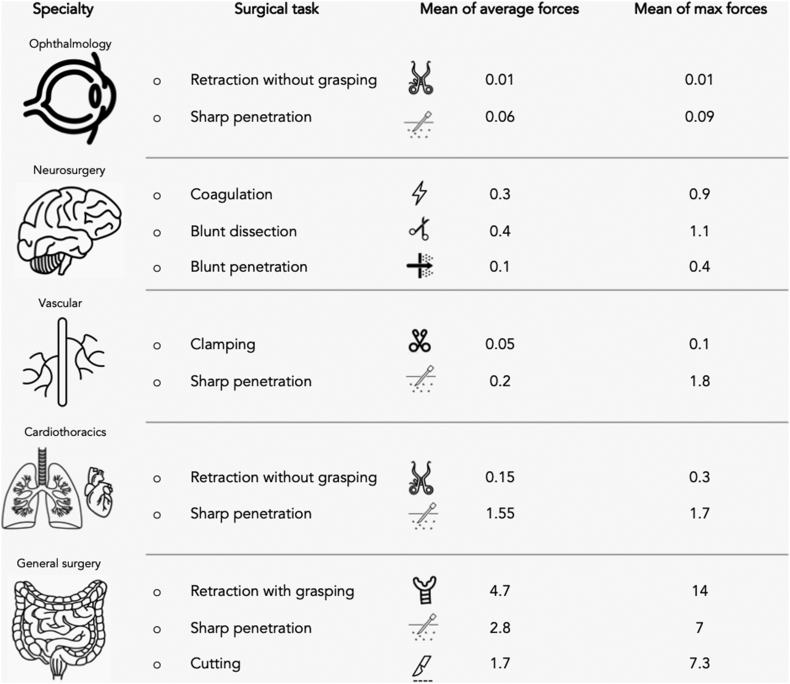
Selected speciality-specific tasks with overview of forces.

Summary forces per speciality are presented in Table 1 and Fig. 2. Generally, across tasks and models, specialities requiring the smallest amount of mean forces were: ophthalmology (mean of average 0.04N) [[17], [18], [19],^21^,^32^], vascular (mean of average 0.07N) [29,40,50], neurosurgery (mean of average 0.68N) [1,2,4,14,22,23,25,28,33,37,44,52] and cardiothoracic surgery (mean of average 1.47N) [26,30,53]. Higher mean of average forces, in ascending order, were seen in general surgery (mean of average 4.67N) [3,5,20,26,27,35,38,[41], [42], [43],^49^,^51^,^54^], otorhinolaryngology (mean of average 8.49N) [36,46], obstetrics and gynaecology (mean of average 8.69N) [39,45], urology (mean of average 9.79N) [24] and orthopaedic surgery (mean of average 210N) [47,48].

### 3.3 Summary forces for tissues systems and surgical manoeuvres

Across specialities and manoeuvres, nervous tissue (principally the brain & retina) required the least amount of force to manipulate (mean of average 0.4N, mean max 1.74N, n = 17) [1,2,4,[17], [19],[21], [22], [23],^25^,^28^,^32^,^33^,^37^,^44^,^52^] with retinal forces always below 0.01N (Table 1) [[17], [18], [19],^21^,^32^]. Mean forces applied on epithelial tissues were higher (mean of average 3.8N, mean max 9.7N, n = 18) [3,14,20,24,26,27,29,35,38,[40], [41], [42], [43],^45^,^50^,^51^,^54^], followed by muscle (mean of average 4.1N, mean max 6.7N, n = 3) [5,49,51], and connective tissue including bone (mean of average 45.8N, mean max 347.9N, n = 10) [20,26,30,31,36,39,[46], [47], [48],^53^]. Mean forces per surgical manoeuvre are reported in Table 1 with drilling recording the highest forces (mean of average 14.1N, mean max 25.9N, n = 2) [46,48], whilst sharp dissection recorded the lowest (mean of average 0.03N, mean max not available, n = 1) [1]. The most recurrent tasks for the top 5 specialities (according to study number) are highlighted in Fig. 2.

### 3.4 Impact of experience and feedback

Eight studies compared novices (e.g. students) to intermediates (e.g. surgical trainees) or experts (e.g. consultant surgeons) (Table 3) [17,19,26,43,46,47,50,55]. When comparing the differences in the pooled mean of average forces exerted by experts and novices, novices exerted 22.7% (range 0–62%, n = 7) more force across tasks and specialities [17,19,26,43,46,47,50,55]. Two studies compared those with intermediate experience to novices and when comparing the differences in the mean of average force, novices applied 29% (range 29-29%, n = 2) more force [19,32]. When comparing experts with intermediates (3 studies), intermediates exerted on average 9.6% (range -50-50%, n = 3) more than experts [19,32,47]. However, in one of these studies experts applied significantly more force (200% of forces applied by intermediates) - with this study detailing forces during revision arthroplasty (screw extraction and implant extraction) where experts may have been more familiar with the significant amount of forces required to remove implants (and therefore less conservative) [47].

**Table 2:** Overview of force feedback mechanism reported.

Study	Feedback groups	Task	Speciality	Mean of average forces pre-feedback	Mean of average forces post-feedback	Absolute Difference	% Difference
Gonenc 2012	Audio (vs. no feedback)	Sharp penetration	Ophthalmology (robotic)	0.1	0.07	0.03	30%
Horeman 2013	Visual force feedback (vs. visual time feedback)	Retraction with Grasping	General surgery (laparoscopic)	0.79 (Visual time feedback)	0.51 (Visual force feedback)	0.28	35.4%
Alleblas 2017	Haptic (vs. no feedback)	Retraction with Grasping	General surgery (laparoscopic)	4.6	1.7	2.9	63%
Diez 2018	Haptic (vs. no feedback)	Multiple (laparoscopic tasks)	Obstetrics and Gynaecology (laparoscopic)	0.81	0.48	0.33	40.7%
Ebrahimi 2018	Haptic (vs. no feedback)	Sharp penetration	Ophthalmology (robotic)	0.12	0.09	0.03	25%
Ebrahimi 2018	Audio (vs. no feedback)	Sharp penetration	Ophthalmology (robotic)	0.12	0.08	0.04	33.3%
Wottawa 2016	Tactile (vs. no feedback)	Retraction with Grasping	General surgery (laparoscopic)	3.4	2.3	1.1	32.4%
Horeman 2012	Visual (vs. no feedback)	Sharp penetration	General surgery (laparoscopic)	2.6	1.3	1.3	50%

**Table 3:** Overview of impact of experience levels on force exertion.

Study	Task	Speciality	*Mean of Average Forces* Novice	*Mean of Average Forces* Intermediate	*Mean of Average Forces* Expert	% Difference Novice Vs Intermediate	% Difference Novice Vs Expert	% Difference Intermediate Vs Expert
Horeman 2012	Sharp penetration	General surgery (laparoscopic)	4.5		2.7		40% more	
Kobler 2015	Drilling	Otolaryngology	13.85		12.29		11% more	
Wottawa 2016	Retraction with Grasping	General surgery (laparoscopic)	3.65		3.15		14% more	
Gonenc 2017	Sharp penetration	Ophthalmology (robotic)	0.05	0.07	0.05	29%	0% more	29% more
Rafii-Tari 2017	Sharp penetration	Vascular surgery	0.32		0.12		62% more	
Talasaz 2017	Sharp penetration	Cardiothoracic surgery (robotic)	2.54		2.49		2% more	
Ebrahimi 2019	Sharp penetration	Ophthalmology (robotic)	0.10	0.14	0.07	29%	30% more	50% more
Kwong 2019	Retraction with grasping	Orthopaedic		470.7	941.4			50% less

Additionally, 7 studies reported effects of feedback mechanisms on forces [5,17,18,32,35,39,43,55]. Feedback mechanisms were haptic (n = 3), visual (n = 2), tactile alone (n = 1) and audio (n = 1) based. Six studies compared feedback to no feedback [5,17,18,32,35,39,55], whilst one study compared visual feedback based on force and visual feedback based on time taken [43]. Pooled mean forces for all feedback mechanisms (across tasks, models and experience level) were reduced by 47.9% (pre-feedback 1.57N, post-feedback 0.8N). Table 2 details reported studies and the mean of average force feedback reports. Two studies aimed to establish the presence of a learning effect by measuring the forces applied before and after being trained on how to perform the task or by performing a series of tasks consecutively, and in both cases the addition of a feedback system (visual or haptic) decreased the applied forces across the learning process when compared to no feedback [5,43]. One study explored the effect of toggling tactile feedback on both hands during a laparoscopic grasper task, finding that average forces exerted by the dominant hand were similar amongst the experts (3.5N, IQR 3–4) and novices (3.5N, IQR: 3–4) without feedback and reduced by tactile feedback to a similar degree (experts 2.3, IQR: 1.4–3.3); (novices 2.2N, IQR: 1.5–2.7) [55]. However, novices applied greater force without tactile feedback with the non-dominant hand (novices 3.8N; experts 2.8N) being reduced again by a similar proportion with tactile feedback (novices by 71.1%; experts by 71.4%). This suggests that non-dominant forces exerted by novices can by reduced with tactile feedback to the levels (median 2.7N, IQR: 1.6–3.8) that experts would apply via the non-dominant hand without tactile feedback (median 2.8N, IQR: 1.8–4) [55].

## 4 Discussion

### 4.1 Principal findings

The measurement of tool-tissue forces during surgery is a new and expanding field, with early adopters including general surgery, neurosurgery and ophthalmology. Through this review, it is clear that the range of forces applied across specialities, tissues, and tasks is highly variable. The highest forces are seen in specialities requiring bony drilling (orthopaedic) and abdominal organ manipulation (urology) [24,47,48]. Whilst lowest forces are seen in specialities encountering more delicate tissue – ophthalmology, neurosurgery and vascular surgery [1,2,4,14,[17], [18], [19],[21], [22], [23],^25^,^28^,^29^,^32^,^33^,^37^,^40^,^44^,^50^,^52^]. There is a relative paucity in data regarding the complications arising from applying too much force. Generally, expert surgeons tend to apply less force than novice and intermediate surgeons, and operators tend to apply less force after repeating the task consecutively [17,19,26,43,46,47,50,55]. A caveat to this was when applying extremes of forces (e.g. arthroplasty implant removal), where senior surgeons tend to apply slightly more forces than novice and intermediate surgeons, perhaps due to their confidence (in applying necessary forces and in handling novel devices) [19,47]. Feedback of any modality (haptic, visual, audio) decreases exerted forces by all users (across experience levels) [5,17,18,32,35,39,43,55].

### 4.2 Findings in the context of literature

The measurement of intraoperative tool-tissue forces may have multiple utilities in the future of surgery. Firstly, in terms of training and assessment, force metrics (average forces, maximum forces, time spent over threshold force, etc) may provide objective data regarding surgeon skill level and therefore may be used as a tool for learning [4,9,10]. Our review contains many studies using phantom models to simulate operations – with tool-tissue force data, these could be refined further and made more high fidelity (for example, designing alerting mechanisms based on established force safety systems) [12,13]. Surgeons could use force data to facilitate reflection on technique and highlighting unconscious excessive force use (e.g. with the non-dominant hand whilst retracting tissue) [12,55]. Using this data to assess the effect of human factors on operative performance (e.g. fatigue, personal stress) could facilitate the development of supportive work environments which optimise surgical care [56,57].

Indeed, examining force data in the context of intra-operative errors and post-operative outcomes may provide an added layer of granularity to this reflection on performance [4]. Establishing the safe thresholds of applicable forces based on tissue type, and building this into operative workflow or devices, may improve the safety of surgery [12]. This is particularly important in laparoscopic and endoscopic surgery, where lack of depth perception, trocar friction, motion scaling, mirroring effects and the size of available force sensors, make it difficult to estimate the forces exerted at the tip of the instruments [58].

Additionally, the development of novel technology will allow measurement and use of intra-operative force data in real-time. Smart force-limiting instruments are one such example, measuring forces and providing feedback based on this to the surgeon [12]. Such instruments have shown potential in decreasing the exerted forces (independently of the surgeon's grade) while not significantly disturbing the flow of the surgical procedure [12]. This technology can be employed in isolated instruments or as part of robotic platforms [59]. Although the sensing of forces within these platforms has been refined, using these forces to provide second-by-second surgeon feedback (particularly haptic) has proven difficult in the current generation of systems used in clinical practice [59,60]. In experimental, ex vivo studies, however, the use of robotic platforms with inbuilt force feedback mechanisms have shown promise in improving surgical performance and increasing surgical safety (e.g. decreased tissue damage) – particularly for less experienced surgeons [55,61].

### 4.3 Limitations and strengths

The main limitation of this review is the heterogeneity of data, making the synthesis and comparison of studies difficult. Reported force values were variable, with some studies using mean/median forces, some maximum forces, and others reporting the root mean square values. Very few studies were matched for tissue or model type (with a relative paucity of in-vivo human studies), or for procedure or task. A standardised data set for reporting, developed by e.g. a consensus process, may prove useful in aligning research going forward. Additionally, the majority of experiments comparing experts and novices assessed each group using the same surgical task (Supplementary material 2) [17,19,26,43,46,47,50,55]. However, when considering future study design and practical translations of comparing experts to novices, recognising that novices and expert surgeons may not complete the same parts of the operation is an important factor to consider. Furthermore, only a minority of included studies were in-human and explored complications related to excess force (e.g. sharp or blunt damage to surrounding tissues or vessels) or insufficient force (e.g. delayed or incomplete task completion), which has clear clinical implications and is an important area for further research [3,4]. Finally, in this review we solely considered the forces measured directly since the models designed to measure forces indirectly were often not validated or they did not report a specific force value. In the future, comparing forces measured directly with those measured indirectly would offer a valuable insight into the accuracy of these models when estimating actual forces from the forces applied by the surgeon to the tool or from the alteration in the tissue shape.

## 5 Conclusion

The measurement of tool-tissue forces during surgery is an expanding field. In the context of heterogeneous data reporting and study design, neurosurgery, ophthalmology and vascular surgery require the least amount of force, whilst orthopaedic surgery required the most. Accordingly, nervous tissue was the most delicate, whilst connective tissue required more force to manipulate. Generally, experts apply less force, and trainees benefit more from force feedback. Looking forward, standardised reporting of tool-tissue interactions will facilitate pooled analysis of force safety thresholds and performance metrics. Development of novel technology such as smart instruments and robotics will facilitate, and benefit from, these advancements.

## Ethical approval

Ethical approval was unnecessary due to the nature of the study (systematic review).

## Author contribution

AKG: data collection, data analysis, manuscript drafting. DZK: study design, data collection, data analysis, manuscript drafting, criitical revisions. GPM: study design, data analysis, manuscript drafting, criitical revisions. HJM: study design, data analysis, manuscript drafting, criitical revisions.

## Research registration number

1. Name of the registry: PROSPERO.
2. Unique Identifying number or registration ID: CRD42020170917.
3. Hyperlink to your specific registration (must be publicly accessible and will be checked): https://www.crd.york.ac.uk/prospero/display_record.php?RecordID=170917.

## Guarantor

George P Mylonas and Hani J Marcus are joint senior authors and guarantors for this study.

## Provenance and peer review

Not commissioned, externally peer-reviewed.

## Patient anonymity and informed consent

Not applicable due to the nature of the study (systematic review).

## Data availability statement

The data that support the findings of this study are available from the corresponding author, upon reasonable request.

## Declaration of competing interest

All authors certify that they have no affiliations with or involvement in any organization or entity with any financial interest (such as honoraria; educational grants; participation in speakers' bureaus; membership, employment, consultancies, stock ownership, or other equity interest; and expert testimony or patent-licensing arrangements), or non-financial interest (such as personal or professional relationships, affiliations, knowledge or beliefs) in the subject matter or materials discussed in this manuscript.
