# Supplementary material for: Tool-tissue forces in surgery: A systematic review
Source: Ann Med Surg (Lond). 2021 Mar 31;65:102268. doi: 10.1016/j.amsu.2021.102268 (PMC8058906; doi:10.1016/j.amsu.2021.102268)
Supplement: Multimedia component 1 [file mmc1.docx]

*Supplementary Information 1: Search strategy used across three scientific databases*

| **PICO format** | **Description** | **Search terms** |
| --- | --- | --- |
| Population | Tissues (natural & synthetic) | (tissue or tissues or vein or veins) |
| Intervention | Instrumented tasks | AND ("tool" OR "tools" OR "device" OR "devices" OR "instrument" OR "instruments") |
|  | Surgery | AND (surgery or surgical or operation or operative)) |
| Control | Not applicable | - |
| Outcome | Interaction forces | AND (force or forces) AND (interaction or interactions) |
