# Supplementary material for: Tool-tissue forces in surgery: A systematic review
Source: Ann Med Surg (Lond). 2021 Mar 31;65:102268. doi: 10.1016/j.amsu.2021.102268 (PMC8058906; doi:10.1016/j.amsu.2021.102268)
Supplement: Multimedia component 2 [file mmc2.docx]

*Supplementary Table 2: Overview of studies included in the final analysis*

| **Author** | **Year** | **Speciality** | **Model: type** | **Model: tissue** | **Tool(s)** | **Procedures** | **Operators** | **Forces: Measurement** | **Forces: Amount** |
| --- | --- | --- | --- | --- | --- | --- | --- | --- | --- |
| Gupta | *1999* | Ophthalmology | Animal cadaver (ex-vivo), human (in-vivo) | Retina (porcine & human) | Retinal pick | Lifting of retina | 6 intermediate  4 novice | Direct force sensors | Retinal manipulation forces:  -75% of mean forces : < 0.01 N  -42.5% of forces: 0 – 0.003 N |
| Howard | *1999* | Neurosurgery | Human (in-vivo) | Brain | -2.5 mm stainless steel sphere attached to a thin stiff wire  -3 mm ventricular shunt catheter | Penetration forces on 2.5-mm spheres and drag forces on 3.0-mm ventricular shunt. Catheters advanced 2.0–3.5 cm deep into the brain (during temporal lobectomy) at rates of 0.33 mm/s. | 1 expert | Direct force sensors | -Range: 0.02 - 0.15 N  -Penetration forces (sphere): average 0.08 N (SD 0.02)  -Drag forces (catheter): average 0.03 N (SD 0.003) |
| Chen | *2004* | Neurosurgery | Phantom (ex-vivo)  Animal (in-vivo) | Brain (agarose gel 0.6%; 0.8%;  porcine) | Silastic 3 mm ventricular shunt catheter | Catheter insertion | 1 expert | Motor-driven force gauge apparatus | Penetration forces ≤ 0.1 N |
| Chmarra | *2004* | General Surgery (Laparoscopic) | VR trainer (ex-vivo) | Bowel | Laparoscopic grasper | -Balls task: putting three balls in three holes in a specified order.  -Ring task: passing a needle through two rings.  -Elastic band task: stretching an elastic band between two rings. | 19 experts | Direct force sensors | -Mean forces across all tasks: 6.8 N  -Force required to prevent slippage of tissue during all tasks: 3.0 N |
| Picod | *2005* | General surgery (Laparoscopic) | Animal (in-vivo) | Porcine bladder, ovaries, uterus, GI tract | Laparoscopic grasper | Laparoscope insertion & removal;  Dissection using laparoscopic grasper | NA | Direct force sensors | Range of forces during insertion–removal of laparoscope:  - Internal pelvic wall: 0.5 -10 N  - Half-voided bladder: 0.4- 3.5 N  - Ovary: 0.2-6 N  Range of forces during dissection:  -Pelvic parietal peritoneum: 0.5-8 N |
| Ye | *2005* | Vascular surgery  (robotic) | Animal (in-vivo) | Rodent aorta | Robotic forceps | Vessel occlusion | 20 novices | Direct force sensors | Minimal occlusion force without causing tissue damage:  -Mean 0.05 - 0.13N, max 0.2N  -Vessel almost fully occluded by 0.05N |
| Ortmaier | *2006* | Orthopaedics  (robotic) | Phantom (ex-vivo), Animal (ex-vivo) | Spine model (solid rigid polyurethane foam)  Porcine Spinal vertebrae | Drill | Robot-assisted drilling & milling of bone (for pedicle screw placement) | NA | Direct force sensors | Max forces during drilling:  -Artificial bone: 15N  -Bovine bone: 15N  Max forces during milling:  -Artificial bone: 12 N  -Bovine bone: 10 N |
| Podder | *2006* | Urology | Humans (in-vivo) | Prostate, perineum | Brachytherapy needles - 17G & 18G | Needle insertion | 2 experts | Direct force sensors | Mean of max forces using 17G needle:  - Perineum: 8.87N (SD 2.32N)  - Prostate: 6.28N (SD 1.64N)  Mean of max forces using 18G needle:  - Perineum: 15.57N (SD 2.98N)  - Prostate: 8.42N (SD 1.52N) |
| Sharp | *2009* | Neurosurgery | Animal (in-vivo) | Brain (mouse) | Cylindrical stainless steel probes 100 & 200 μm flat punch probes; sharpened tip probe | Probe penetration | NA | Direct force sensors | Range of forces by tissue type:  -Olfactory bulb: 0.32 - 1.61 N  -Cortex: 0.51 - 2.48 N |
| Bell | *2010* | Otorhinolaryngology | Cadaver (ex-vivo) | Excised incus  temporal bone specimen | Micro-forceps | Stapedectomy | 1 expert | Direct force sensors | Max forces range: 2.4 N-5.2 N |
| Yip | *2010* | Cardiothoracic surgery | Animal (in-vivo) | Porcine mitral valve | Needle – 14G | Mitral valve annuloplasty (securing anchor to valve annulus) | NA | Direct force sensors | Passive forces whilst instrument stationary: 0 - 0.3 N  Anchor deployment:1.5 N |
| Trejos | *2011* | General surgery | Animal (in-vivo) | Porcine uterine horns (mimic appendix) | Grasping forceps;  Endoscopic scissors | Trans-gastric & trans-perineal appendectomy | 2 experts | Direct force sensors | Trans-gastric procedure: mean 0.80 ±1.1 N, max 7.9N: -Pull = 0.53 ± 0.67; max 7.9 -Push in = 0.46 ± 0.52, max 2.9 -Push sideways 0.82 ± 0.64; max 3.9 -Cut 2.8 ± 1.7; max 6.1  -Orient appendix: 0.55 ± 0.84; max 7.8  -Mobilize mesoappendix:0.67 ± 0.71; max 7.3  -Cut endoloop: 2.8 ± 1.7; max 6.1  -Divide appendix: 0.61 ± 0.57; max 7.9  Trans-perineal procedure: mean 1.4 ± SD (1.4 N), max 15.8 N  -Pull 1.9 ± 1.3; max 15.8  -Push in 1.7 ± 1.3; max 7.8  -Push sideways 1.9 ± 0.76; max 6.8  -Cut 0.69 ± 0.66; max 4.1  -Orient appendix: 0.61 ± 0.57; max 11.9  -Mobilize mesoappendix: 1.7 ± 1.2; max 15.8  -Cut endoloop: 0.69 ± 0.66; max 4.2  -Divide appendix: 2.4 ± 1.7; max 6.6 |
| Famaey | *2012* | Vascular surgery | Animal (in-vivo) | Rodent aorta | Clamping forceps | Vessel occlusion | NA | Direct force sensors | Minimum occlusion forces:  - Flat edge clamp: 0.001 N  - Smooth edge clamp: 0.005 N |
| Gonenc | *2012* | Ophthalmology (robotic) | Animal (ex-vivo) | Shell membrane from raw chicken eggs (simulating retinal membrane) | Hand-held tremor-cancelling micromanipulator (Micron) | Vitreoretinal microsurgery (membrane peeling) | 1 novice | Direct force sensors | -Safety threshold before rupture < 0.007 N  -Removal of tremor cancelling and audio force feedback resulted in forces > 0.01 N |
| Horeman | *2012* | General surgery  (Laparoscopic) | Phantom (ex-vivo) in laparoscopic box trainer | Artificial skin | Needle | Suturing (two-handed needle driving & knot tying) | 11 experts  21 novices | Direct force sensors | Needle driving:  -Novice mean max: 4.5 N (SD: 1.3)  -Novice mean of mean *NZ: 1.6 N (SD: 0.6)  -Experts mean max: 2.7 N (SD: 0.4)  -Experts mean of mean NZ: 0.9 N (SD: 0.3)  Knot tying:  -Novice mean max: 4.3 N (SD: 0.9)  -Novice mean of mean NZ: 0.5 N (SD: 0.2)  -Expert mean max: 2.7 N (SD: 1.2)  -Expert mean of mean NZ: 0.4 N (SD: 0.1)  Average Max force peak (time x pulling force)  -Experts 6.7 N (SD: 7.7)  -Novice 15.4 N (SD: 10.5)  *NZ is a direction in which force was recorded. |
| Horeman | *2012* | General surgery  (Laparoscopic) | Phantom (ex-vivo) in laparoscopic box trainer | Artificial tissue (elastic) | Needle | Needle driving with laparoscopic grasper | 12 novice | Direct force sensors | Average Force post task training  -With Visual feedback: 1.3 (SD: 0.6)  -No feedback: 2.6 (SD: 0.9)  Max Force post task- training  -With visual feedback 4.1N (SD: 1.1)  -No feedback: 8N (SD: 3.3) |
| Rodrigues | *2012* | General surgery | Animal (ex-vivo) | Porcine aorta, Vena Cava, GI tract, Uterus, Fallopian tube | Needle | Suturing | NA | Direct force sensors | Average max acceptable (safe) force before tissue damage:  -Fascia: 11.5N  -Aorta: 9.3N  -Vena Cava: 4.7N  -Peritoneum: 1.8N  -Large bowel: 2.1N -Uterus: 3.2N  -Fallopian tube: 1.2N |
| Goyzueta | *2013* | General surgery | Animal (ex-vivo) | Porcine liver | Grasping forceps: flexible & rigid | Grasping  Pulling (whilst graspers closed) | NA | Force sensitive resistor (FSR) | Median Maximum forces  -Grasping with rigid grasper: 8.7N -Pulling with rigid grasper: 8.1N (SD: 0.8)  -Pulling with compliant grasper: 1.4N (SD: 0.6) |
| Horeman | *2013* | General surgery  (Laparoscopic) | Phantom (ex-vivo) in laparoscopic box trainer | Artificial tissue (elastic) | Laparoscopic grasper | Tissue manipulation | 25 novices | Direct force sensors | Mean Absolute Force  -With visual time feedback*: 0.79 (0.39)  -With visual force feedback^+^: 0.51 (0.19)  Max Absolute Force  -With visual time feedback: 9.33 (3.96)  -With visual force feedback**:** 6.68 (1.61)  Comparison between first and last repetition:  -Mean Absolute Force: with visual force feedback= 0.43N; with time feedback= NA  -Maximum Absolute Force: with visual force feedback= 3.27 N; with time feedback= 2.56N  *Time feedback: after a set time a recorded voice would remind the user how much time they has spent manipulating that tissue. ^+^Force feedback: if excessive force was applied, a red arrow would be displayed as overlay onto the box trainer screen, pointing toward the area of high force exertion |
| Talasaz | *2013* | General surgery  (Laparoscopic) | Animal (ex-vivo) | Bovine liver (tumour) | Tissue probe | Tissue palpation & tumour localization | 1 expert 3 intermediate 4 novice | Direct force sensors | Mean forces:  -Palpation to locate tumour: 4 - 5N.  -Lateral movement (moving the probe right and left to locate the tumour): -0.5 - 0.5N. |
| Marcus | *2014* | Neurosurgery  (robotic) | Cadaver (ex-vivo) | Brain | Robotic tele-operated arm | -Stab incision  -Carrying incision  -Retraction  -Arachnoid dissection | NA | Direct force sensors | Median forces by task:  -Arachnoid dissection: 0.22 N  -Sharp dissection: 0.03 N  Median forces by tissue type:  -Rectus gyrus  -Stab incision < 0.01; Carrying incision 0.02; Retraction 0.03  -Inferior Temporal gyrus  -Stab incision < 0.01; Carrying incision 0.02; Retraction 0.07  -Middle Frontal gyrus  -Stab incision < 0.01; Carrying incision 0.15; Retraction 0.08  -Cerebellar hemisphere  -Stab incision 0.01; Carrying incision 0.03; retraction 0.08  -Cerebellar vermis  -Stab incision 0.02; Carrying incision 0.12; Retraction Na  -Midbrain  -Stab incision 0.01; Carrying incision 0.1; Retraction 0.15  -Pons  -Stab incision < 0.01; Carrying incision 0.05; Retraction 0.18  -Medulla  -Stab incision 0.01; Carrying incision 0.09; Retraction 0.09  -Corpus Callosum  -Stab incision 0.01; Carrying incision 0.23; Retraction NA  -Perforating floor of 3^rd^ ventricle  -Stab incision < 0.01; Carrying incision NA; Retraction NA |
| Gan | *2015* | Neurosurgery | Cadaver (ex-vivo) | Brain | Bipolar forceps | -Coagulation  -Dissection | 1 expert  1 novice | Direct force sensors | Dissection forces by vessel type:  -Small cortical vessels  -Mean peak: 0.2 to 0.28; min: 0.13 to 0.29; max: 0.17 to 0.40  -Middle cerebral artery  -Mean peak: 0.30 to 0.34; min: 0.09 to 0.42; max: 0.80 to 0.85  -Sylvian fissure  -Mean peak: 0.42 to 0.56; min: 0.11 to 0.58; max: 0.63 to 0.83  -Interhemispheric fissure  -Mean Peak: 0.38 to 0.52; min: 0.12 to 0.28; max: 0.7 to 0.89  -Optic chiasm (optic n. & carotid artery)  -Mean Peak: 0.5 to 0.59; min: 0.2 to 0.21; max: 0.95 to 1.18  -Oculomotor nerve  -Mean Peak: 0.41 to 0.55; min: 0.19 to 0.28; max: 0.57 to 0.82  Coagulation forces by vessel type:  -Scalp vessel  -Mean Peak: 0.22 to 0.32; min: 0.05 to 0.075, max: 0.51 to 0.85  -Pia/arachnoid over temporal gyrus  -Mean Peak: 0.09 to 0.19; min: 0.04 to 0.11; max: 0.35 to 0.54  Dissection and division forces of white matter by tissue type:  -Temporal stem  -Mean Peak: 0.11 to 0.12; Min: 0.01 to 0.09; Max : 0.34 to 0.45  -Corpus callosum  -Mean Peak: 0.2 to 0.4; Min: 0.05 to 0.18; Max: 0.54 to 0.79  Coagulation forces by tissue type:  -Temporal stem  -Mean Peak: 0.26 to 0.33; Min: 0.04 to 0.06; Max: 0.63 to 0.83  -Corpus callosum  -Mean Peak: 0.32 to 0.345; Min: 0.04 to 0.09; Max:1.03 to 1.13 |
| Kobler | *2015* | Otorhinolaryngology | Phantom (ex-vivo) | Synthetic Cochlea (Solid Rigid Polyurethane, homogenous bone substitute) | Drill | Cochlear drilling | 1 expert  1 novice | Direct force sensors | Maximum forces during drilling:  -Expert: 6.49 (x-axis), 9.13 (y-axis), -21.26 (z-axis)  -Novice: 25.99 (x-axis), 5.85 (y-axis), 11.65 (z-axis), 11.89 (y-axis) |
| Sutherland | *2015* | Neurosurgery  (robotic) | Humans (in-vivo) | Brain (tumour) | Bipolar forceps via NeuroArm | Glioma resection | NA | Direct force sensors | Majority of forces: < 0.6 N  Max total force: 2.45 N  Average Forces (N)  - X-axis: 1.37N  - Y-axis: 1.84N  - Z-axis: 2.01N |
| Aggravi | *2016* | Neurosurgery | Phantom (ex-vivo) | Synthetic brain (7% gelatin & water) | Bipolar forceps  Dissector  Suction  Spatula | -Texture task: touching the brain model surface with bipolar forceps, spatula and suction.  -Penetration task: 10 mm straight insertion of bipolar forceps 20mm & straight insertion of suction tool into brain model. | 3 experts | Direct force sensors | Texture task: max 0.01 N Penetration task: max: 0.59 N |
| Barrie | *2016* | General surgery  (Laparoscopic) | Animal (in-vivo) | Porcine gallbladder, bladder, rectum, large bowel & small bowel | Laparoscopic grasper (short fenestrated) | -Grasping task for all organs;  -Running small bowel: alternately passing small bowel between right- and left-handed grasper. | 1 expert | Direct force sensors | Grasping task: mean of max forces + SD & mean of RMS*   - Colon: max 59 (SD: 13.4 N), RMS 24.6N - Gallbladder: max 50.7 (SD: 3.8N), RMS 24.3N - Rectum: max 49 (SD: 15N), RMS 21.4N - Bladder: max 28.8 (SD: 7.4N), RMS 21.9N   Running small bowel: mean of max 20.5N (SD: 7.2), RMS 9.7N  *RMS = root mean square force (average force across 30 s of grasp time) |
| Maddahi | *2016* | Neurosurgery  (robotic) | Human (in-vivo) | Brain (tumour) | Bipolar forceps on NeuroArm  Suction on NeuroArm | Resection of tumours (low & high grade gliomas) – during use of bipolar forceps and suction | 1 expert | Direct force sensors | Mean forces using bipolar forceps:  - X-axis: 0.17N (SD: 0.01)  - Y-axis: 0.18N (SD: 0.02)  - Z-axis: 0.22N (SD: 0.05)  - F-axis: 0.38N (SD: 0.05)  Mean forces using suction:  - X-axis: 0.09N (SD: 0.01)  - Y-axis: 0.10N (SD: 0.01)  - Z-axis: 0.13N (SD: 0.02)  - F-axis: 0.21N (SD: 0.03)  Mean of peak forces across all instruments  - X-axis: 1.67N  - Y-axis: 1.65N  - Z-axis: 1.68N  - F-axis: 1.86N |
| Maddahi | *2016* | Neurosurgery | Cadaver (ex-vivo) | Brain (normal tissue) | Bipolar forceps | Vessel dissection & coagulation | 1 expert (+ assistant) | Direct force sensors | Mean forces during dissection & coagulation:  -X-axis: 0.9 ± 0.3N -Y-axis: 1,2 ± 0.5N  -Z-axis: 1.3 ± 0.8N  Max forces during dissection & coagulation:  -X-axis: 1.4N  -Y-axis: 2.9N  -Z-axis: 3N |
| Wang | *2016* | Neurosurgery | Cadaver (ex-vivo) | Brain | Bipolar forceps | Vessel dissection & coagulation | 1 expert,  1 intermediate | Direct force sensors | Average forces across all tasks:  -1.94 N for superior cerebellar artery  -1.75 N for middle cerebellar artery |
| Zareinia | *2016* | Neurosurgery | Cadaver (ex-vivo) | Brain | Bipolar forceps | Vessel dissection | 1 expert | Direct force sensors | Dissection forces by tissue type:  -Lilliquist’s membrane: mean 0.35 N, max 0.95 N  -Carotid cistern: mean 0.41 N, max 0.97 N  -Basilar bifurcation: mean 0.48 N, max 1.07 N  -Pericallosal artery: mean 0.46 N, max 1.15 N  -Opening ependyma into lateral ventricle: mean 0.37 N, max 0.82 N |
| Wottawa | *2016* | General Surgery (Laparoscopic surgery, robotic) | Animal (in-vivo) | Bowel (porcine) | Laparoscopic grasper on Da Vinci robot with deactivated & activated tactile feedback | Tissue grasping | 5 experts,  14 novices | Direct force sensors | Median forces (IQR):  -Dominant hand  -Novice: 3.5 (3.0 – 4.0); with tactile feedback: 2.2 [1.5 – 2.7]  -Expert: 3.5 [3.0 – 4.0]; with tactile feedback: 2.3 [1.4 – 3.3]  -Non-dominant hand  -Novice: 3.8 [1.8 – 4.1], with tactile feedback: 2.7 [1.6 – 3.8]  -Expert: 2.8 [1.8 – 4.0], with tactile feedback: 2.0 [1.5 – 2.3] |
| Alleblas | *2017* | General surgery  (Laparoscopic) | Animal (ex-vivo) in laparoscopic box trainer | Porcine  (lung, small intestine & liver) | Laparoscopic graspers:  force-sensing grasper with enhanced haptic feedback,  force-sensing grasper with enhanced haptic feedback deactivated | Tissue discrimination via palpation (operator blind to tissue type) | 7 experts  13 novices | Direct force sensors | Mean forces:  -With haptic feedback: 1.7 N (SD: 0.7)  -Without haptic feedback: 4.6 N (SD:1.5) |
| Gonenc | *2017* | Ophthalmology  (robotic) | Phantom (ex-vivo) | Synthetic retina (vinyl layer stretched onto an acrylic insert with laser cut slots) | Motorized Force-Sensing Microneedle Integrated with a Hand-held tremor-cancelling micromanipulator (Micron) | Robot assisted retinal vein cannulation | 1 expert 1 intermediate  1 novice | Direct force sensors | Membrane puncture  -Range: 0.01 – 0.02 N  -Max: 0.01 – 0.014 N  Rate of force drop at the instant of piercing phantom membrane  -Overall range: −0.04 to −0.01 N/s  -Novice: − 0.05 ± 0.02 N/s  -Intermediate: − 0.07 ± 0.03 N/s  -Expert: −0.05 ± 0.02 N/s |
| Javaux | *2017* | Obstetrics & Gynaecology | Phantom (ex-vivo) | Placenta | Fetoscope | Insertion, coagulation, retraction | 3 experts | Direct force sensors | Median of max forces by instrument configuration:  -Rigid tip  -Central placenta: 8.91 (IQR: 8.34 to 9.34)  -Left placenta: 9.76 (IQR: 9.26 to 10.23)  -Right placenta: 10.1 (IQR: 9.4 to 10.7)  -Flexible tip  -Central placenta: 6.78 (IQR: 6.14 to 8.49)  -Left placenta: 7.72 (IQR: 7.41 to 10.31)  -Right placenta: 8.89 (IQR: 8.62 to 10.49) |
| Rafii-Tari | *2017* | Vascular surgery | Phantom (ex-vivo) | Synthetic aneurysms, tortuous arteries (silicon) | Endovascular catheter | Cannulation & endovascular catheter navigation | 6 expert  10 novice | Direct force sensors | Cannulation:  - Experts: mean 0.11 - 0.13N, max 0.54 - 0.84N (depending on vessel)  - Novices: mean 0.24 - 0.39N, max 1.4 - 1.75N (depending on vessel)  Median of median forces by vessel location and expertise:  -Descending aorta  -Expert: 0.03 N, (IQR: 0.03 to 0.04)  -Novice 0.06 N (IQR: 0.06 to 0.07)  -Arch Vessel  -Expert: 0.09 N (IQR: 0.08 to 0.09)  -Novice: 0.17 N (IQR: 0.15 to 0.18) |
| Sugiyama | *2017* | Neurosurgery | Humans (in-vivo) | Brain (Arteriovenous Malformation) | Bipolar forceps | Arteriovenous malformation resection | 1 expert | Direct force sensors | Coagulation forces (N) by tissue type:  - Scalp vessel: mean 1.03 (0.23), max 1.70 (0.33).  - Dura: mean 0.34 (0.14), mean max 0.43 (0.18)  - Pia-arachnoid: mean 0.27 (0.10), mean max 0.40 (0.19)  - Major feeder and drainer: mean 0.33 (0.11), mean max 0.47 (0.16)  - Small vessel around nidus: mean 0.23 (0.06), mean max 0.35 (0.12)  - Division of gliotic layer between brain and nidus: mean 0.23 (0.06), max 0.34 (0.10) |
| Sumer | *2017* | General surgery  (Laparoscopic) | Phantom (ex-vivo), Animal (ex-vivo) | Synthetic tissue (Polydimethylsiloxane PDMS; silicone rubber)  Animal liver, Muscle | Laparoscopic grasper | Grasping tissue | NA | Direct force sensors | Overall range of forces: 0.02–0.07 N  Max forces by material: -PDMS: 0.07 N  -Silicon rubber: 0.05 N  -Liver :0.02 N  -Muscle: 0.03 N |
| Talasaz | *2016* | Cardiothoracic surgery  (robotic) | Phantom (ex-vivo) | Synthetic mitral valve | Needle on DaVinci arm | Suturing | 6 experts  7 novices | Direct force sensor | Tissue puncturing: mean forces  -Novice: 2.54 N (SD: 0.17)  -Experts: 2.49 N (SD: 0.28)  Knot tightening: mean forces  -Novice: 3.57 N (SD: 0.19)  -Experts: 3.17 N (SD: 0.39) |
| Sugiyama | *2018* | Neurosurgery | Humans (in-vivo) | Brain (vascular malformations & aneurysms, tumours, normal brain) | Bipolar forceps | Vessel dissection & coagulation, dissection of tumour. | 5 expert 5 intermediate  6 novice | Direct force sensors | Summary of *most* intracranial tasks:  -Mean < 0.6N  -Max < 1N  Mean forces across all tasks:  -Novice: 0.74N  -Intermediate: 0.00 N  -Experts: 0.08N  Dissection of small cortical vessel (p<0.001)  -Intermediate 1.31N  -Expert: 0.00 N  Coagulation of small cortical vessel (p=0.02)  -Intermediate 0.54N  -Expert 0.00N  Division of glioma (p= 0.01)  -Intermediate -0.5N  -Expert 0.00 N  Force required to divide brain:  -Mean 0.45 - 0.54N  -Max 0.71N - 0.84N |
| Diez | *2018* | Obstetrics and Gynaecology  (Laparoscopic) | Phantom (ex-vivo) | Tomato (model of endometrium) | Laparoscopic grasper | Grasping, peeling and ablating exocarp from mesocarp. | NA | Direct force sensors | Average maximum force:  -No haptic feedback: 6.87 (SD: 4)  -Haptic feedback: 4.28 (SD: 1.96)  Average force:  -No haptic feedback: 0.81 (SD: 0.42)  -Haptic feedback: 0.48 (SD: 0.3) |
| Ebrahimi | *2019* | Ophthalmology | Phantom (ex-vivo) | Synthetic sclera (silicon) | 25-gauge nitinol needle via SteadyHand Eye Robot | Retinal vessel tracing | 3 experts | FBG optical strain sensors | With active control*:  Median of mean: 0.07 N (IQR: 0.06 – 0.07 N)  Median of max: 0.05 N (IQR: 0.05 – 0.07 N)  With passive control*:  Median of mean: 0.08 N (IQR: 0.07 – 0.09 N)  Median of max: 0.07 N (IQR: 0.06 – 0.08 N)  *Active control = automatic force reduction by robotic platform when force exceeds safe limits.  *Passive control = alarm on reaching force safety threshold. |
| Ebrahimi | *2019* | Ophthalmology  (robotic) | Phantom (ex-vivo) | Synthetic sclera (silicon) | 25-gauge nitinol needle via SteadyHand Eye Robot | -Robot assisted retinal vein-cannulation  -Retinal vessel tracing | 2 novices  1 intermediate  1 expert | FBG optical strain sensors | Overall mean forces  -Without feedback: 0.10 N  -With audio feedback: 0.07 N  Mean forces with no robotic assistance  -Novices: 0.10 N; 0.08 N with audio feedback  -Intermediate: 0.14 N; 0.08 N with audio feedback  -Expert: 0.07 N; 0.06 N with audio feedback  -All : 0.10 N; 0.07 N with audio feedback  Mean forces robotic assistance:  -Novices: 0.12 N, 0.07 N with audio feedback; 0.09 N with haptic feedback  -Intermediate: 0.07 N, 0.10 N with audio feedback; 0.11 N with haptic feedback  -Expert: 0.16 N, 0.10 with audio feedback; not performed with haptic feedback  -All: 0.12 N, 0.08 N with audio feedback; 0.09 N with haptic feedback |
| Kwong | *2019* | Orthopaedics | Phantom (ex-vivo) | Hemipelvis (composite, polyurethane foam) | Acetabular Revision System (Zimmer Vs EZout) | Revision arthroplasty:  Acetabular removal (screw extraction, implant extraction) | 1 expert  1 intermediate | Direct force sensor | Forces during screw extraction:  -Intermediate: mean 98.1(19.6), min 68.6, max 3402.9  -Expert: mean 117.7 (19.6), min 68.6, max 137.3  Forces during implant extraction:  -Intermediate: mean 470.7 (88.3), min 382.5, max 578.6  -Expert: mean 941.4(196.1), min 647.2, max 1206.2 |
| Stoiber | *2019* | Cardiothoracic surgery | Phantom (ex-vivo) | 4 aortic valve types (bioprosthetic, artificial pericardial tissue, fresh porcine, fixated porcine) | Transvalvular cannula,  Catheter-based cannulas or pumps | Insertion of transvalvular cannulas during transcatheter aortic valve implantation: | NA | Force transducer | Maximum forces by location:  -Middle of cusps: 0.8 N  -Commissures: 0.2 N  Maximum forces by tissue type:  -Fresh porcine valve: 0.40 N  -Fixated porcine valve: 0.56 N  -Artificial pericardial valve: 0.75 N  -Bioprosthetic valve: 0.78 N |
| Hardon | *2019* | General surgery  (Laparoscopic) | Phantom (ex-vivo) in laparoscopic box trainer | Bowel (synthetic) | Needle | Purse-string suture during trans-anal total mesorectal excision | 7 novices | Direct force sensors | Range of forces: 2.5 - 4.1 N  Safety threshold for tissue manipulation: 3 N |
