# Supplementary material for: Tool-tissue forces in surgery: A systematic review
Source: Ann Med Surg (Lond). 2021 Mar 31;65:102268. doi: 10.1016/j.amsu.2021.102268 (PMC8058906; doi:10.1016/j.amsu.2021.102268)
Supplement: Multimedia component 3 [file mmc3.docx]

*Supplementary Information 3: Number of articles published by year*
